# Animal Models for DOHaD Research: Focus on Hypertension of Developmental Origins

**DOI:** 10.3390/biomedicines9060623

**Published:** 2021-05-31

**Authors:** Chien-Ning Hsu, You-Lin Tain

**Affiliations:** 1 Department of Pharmacy, Kaohsiung Chang Gung Memorial Hospital, Kaohsiung 833, Taiwan; cnhsu@cgmh.org.tw; 2 School of Pharmacy, Kaohsiung Medical University, Kaohsiung 807, Taiwan; 3 Department of Pediatrics, Kaohsiung Chang Gung Memorial Hospital and Chang Gung University College of Medicine, Kaohsiung 833, Taiwan; 4 Institute for Translational Research in Biomedicine, Kaohsiung Chang Gung Memorial Hospital, Chang Gung University College of Medicine, Kaohsiung 833, Taiwan

**Keywords:** animal model, developmental origins of health and disease (DOHaD), hypertension, oxidative stress, pregnancy, renin-angiotensin system, gut microbiota, reprogramming

## Abstract

Increasing evidence suggests that fetal programming through environmental exposure during a critical window of early life leads to long-term detrimental outcomes, by so-called developmental origins of health and disease (DOHaD). Hypertension can originate in early life. Animal models are essential for providing convincing evidence of a causal relationship between diverse early-life insults and the developmental programming of hypertension in later life. These insults include nutritional imbalances, maternal illnesses, exposure to environmental chemicals, and medication use. In addition to reviewing the various insults that contribute to hypertension of developmental origins, this review focuses on the benefits of animal models in addressing the underlying mechanisms by which early-life interventions can reprogram disease processes and prevent the development of hypertension. Our understanding of hypertension of developmental origins has been enhanced by each of these animal models, narrowing the knowledge gap between animal models and future clinical translation.

## 1. Introduction

The association between fetal development and the increased risk of adult disease has attracted a great deal of attention to the concept of developmental programming or developmental origins of health and disease (DOHaD) [1,2]. The DOHaD hypothesis gained momentum after the emergence of observational studies from the 1944–1945 Dutch famine cohort, illustrating that maternal starvation is associated with an increased risk of metabolic and cardiovascular diseases in adult offspring [3]. These findings, combined with numerous subsequent epidemiologic investigations, indicate that the perinatal period, a critical window of organogenesis, is a vulnerable time in terms of the impact of adverse environmental insults [4]. Several hypotheses, such as thrifty phenotype [5], maternal capital [6], and predictive adaptive responses [7], have been developed to explain the epidemiological observations of an association between early life insults and diseases in adulthood. However, these hypotheses do not propose mechanistic pathways by which disease proceeds or suggest potential interventions for the prevention of adult diseases. Accordingly, animal models that have been developed and characterized have been instrumental in indicating the biological plausibility of the associations observed in epidemiological research, providing proof of causality. Emerging evidence indicates that animal models are valuable tools for understanding the pathogenesis of developmental programming and developing therapeutic interventions for DOHaD-related diseases [8,9,10]. A variety of small (e.g., rats, mice, and guinea pigs) and large (e.g., sheep and pigs) animals have been used to test aspects of the DOHaD hypothesis, and each offers different advantages.

Hypertension and related cardiovascular diseases are leading causes of mortality worldwide [11]. The WHO reported that 1 in 4 men and 1 in 5 women have hypertension [12]. Due to the multifactorial nature of hypertension, the use of various animal models, which induce hypertension by various mechanisms and produce the same end result, is advantageous [13,14]. In the past decades, novel drug classes and interventional strategies for the treatment of hypertension have been developed using hypertensive animal models [15]. However, the prevalence of hypertension remains high and continues to increase globally [16]. All this raises the question of how to prevent and not just treat hypertension based on the DOHaD concept.

A broad range of early-life insults can induce developmental programming, resulting in hypertension. These include maternal undernutrition or overnutrition, maternal disease states, lifestyle changes, substance abuse, environmental exposure to toxins/chemicals, and medication use during pregnancy [10,17,18,19,20]. Hypertension, diabetes, kidney disease, and inflammation are common maternal diseases that complicate pregnancy. On the other hand, programming processes geared toward disease could be reversed by shifting therapy from adulthood to the perinatal period, that is to say, by reprogramming [21]. Although the pathogenesis behind hypertension of developmental origins is poorly understood at present, our understanding of animal models used to study common mechanistic pathways has advanced greatly in recent years, which helps in developing efficient strategies to reprogram hypertension and prevent it from happening.

This review summarizes the contributions of animal models to DOHaD research with a focus on hypertension. It is proposed that integrating evidence from diverse animal models is essential in order to advance our understanding of hypertension of developmental origins and develop novel reprogramming strategies to alleviate the global burden of hypertension.

We retrieved related literature from all articles indexed in PubMed/MEDLINE. Search terms were as follows: “blood pressure”, “developmental programming”, “DOHaD”, “animal model”, “mother”, “maternal”, “pregnancy”, “gestation”, “offspring”, “progeny”, “prenatal”, “perinatal”, “reprogramming”, and “hypertension”. Additional studies were then selected and assessed based on appropriate references in eligible papers. The last search was conducted on 20 April 2021.

## 2. Choice of Animal Models

**Figure 1:**
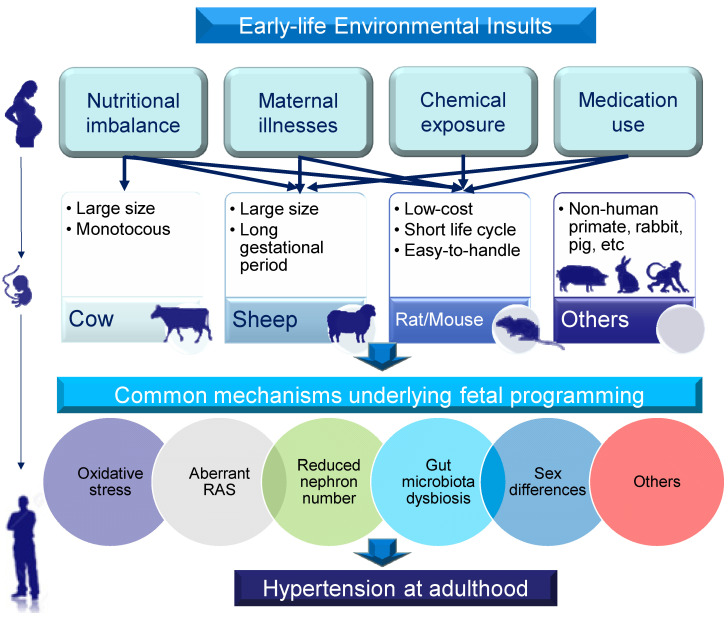
Schematic illustration of the selection of animal models for studying hypertension of developmental origins in adulthood according to early-life environmental insults, animal species, and common mechanisms. Lines with arrows (top section) indicate types of early-life insults produced in particular species of animals to induce hypertension in adult offspring. The study of other animals in DOHaD research (non-human primates, rabbits, pigs, etc.) is limited.

A broad range of animal models have been established to validate that the associations found in human observational studies can be replicated under experimental conditions. Animal models can be categorized in many different ways. First, models for DOHaD research can be categorized by types of environmental insult. For example, global caloric restriction and protein restriction in animals can mimic the starvation associated with famine in human cohorts [8,9]. Second, animal models can be classified according to molecular mechanisms. Since different environmental insults during pregnancy and lactation produce similar outcomes with respect to hypertension in adult offspring, there might be common mechanisms behind the developmental programming of hypertension. To date, hypertension of developmental origins has been attributed to mechanisms [10,17,18,19,20,21] including reduced nephron number, oxidative stress, an aberrant renin–angiotensin system (RAS), gut microbiota dysbiosis, and sex differences, among others. Animal models have been developed to test such proposed mechanisms. Finally, various small- and large-animal models have been established for DOHaD research, each with its own natural advantages and disadvantages [8]. Although non-human primates have long been regarded as the gold standard because of their high genetic and biological similarity to humans, the most commonly used species in the DOHaD field are rodents [22]. Rat and mouse models provide a low-cost option with a short life cycle that is easy to handle. Mice also provide ample access that allows for genetic modification. Depending on the experimental approach, other species such as rabbits, sheep, and pigs have also been used to evaluate developmental programming related to offspring outcomes [22]. Rabbits are useful for studies as their lipid metabolism and placental structure are similar to those in humans [23]. Pigs are considered to be a suitable model for evaluating the early stages of fertilization and development. Sheep have a long gestation period, and their fetal size and developmental rate are close to those in humans [24]. Cows are large, monotocous animals with a long gestation period, as in humans [24]. Thus, many aspects of animal models have to be taken into consideration when choosing one species over another, such as genetic background, anatomy, physiology, length of gestation, litter size, life cycle, and application to the clinical context. A summary of the selection of animal models for the study of hypertension of developmental origins is depicted in Figure 1.

## 3. Hypertension of Developmental Origins: Early-Life Insults

Several suboptimal environmental conditions during fetal development are relevant to hypertension in adult offspring, including maternal nutritional imbalance, maternal illnesses and conditions, exposure to environmental chemicals, and medication use during pregnancy and lactation [10,17,18,19,20]. Each category is discussed in turn.

### 3.1. Maternal Nutritional Imbalance

**Figure 2:**
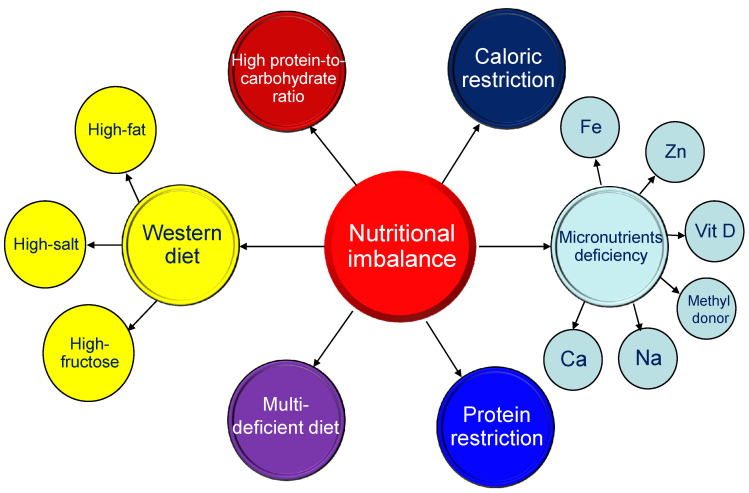
Overview of nutritional interventions used to modulate nutritional status during pregnancy and/or lactation to study hypertension of developmental origins in animal models. Fe = iron; Zn = zinc; Vit D = vitamin D; Na = sodium; Ca = calcium.

Within the context of DOHaD research, studies of nutritional programming using small animal models have been ongoing since the early 1990s [8]. Nutritional interventions during critical developmental phases can have long-lasting effects on blood pressure (BP) in adult offspring [17]. Excessive or insufficient consumption of a specific nutrient has been used to induce hypertension of developmental origins in animal models, as shown in Figure 2 [25].

Caloric restriction refers to an overall reduction of energy and nutrient intake without incurring malnutrition. Caloric restriction in a range of 30–70% in pregnant rats has been reported to induce elevated BP in their adult offspring [26,27,28]. Hypertension programmed by maternal caloric restriction has also been observed in other species, including sheep [29,30] and cows [31]. In general, more severe caloric restriction resulted in earlier development of hypertension in adult offspring [25]. The protein restriction model has also been widely used to explore the mechanisms of nutritional programming [32]. As in the caloric restriction model, when pregnant rats were exposed to a greater degree of protein restriction, their adult offspring were likely to have high BP earlier [33,34,35]. Moreover, deficiencies in micronutrients, including iron [36], zinc [37], vitamin D [38], methyl donor nutrients (folic acid; choline; methionine; and vitamins B2, B6, and B12) [39], sodium [40], and calcium [41] in pregnant rats were associated with hypertension in their offspring. In a Brazilian study, when dams were fed with a multi-deficient diet developed from a basic regional diet, this was also shown to induce hypertension in adult rat offspring [42,43]. On the other hand, the excessive intake of certain nutrients can result in programmed hypertension in male adult offspring [25]. The Western diet is a modern dietary pattern characterized by the high intake of high-fat products, high-sugar drinks, and excess salt. In animal models of maternal diets containing key components based on the human Western diet, synergistic effects of fat, sugar, and salt on the rise of BP in adult progeny were observed [44,45,46]. The most frequently used model to induce obesity-related disorders is a high-fat diet [47]. The BP of adult offspring exposed to a maternal high-fat diet varies according to age, sex, diverse fatty acid composition, and strain [48,49,50]. Similarly, the intake of solely a high-fructose diet by rodent mothers results in BP elevation in the offspring [51,52,53]. A maternal high-fructose diet was developed into an animal model frequently used for studying hypertension and metabolic syndrome of developmental origins [54]. Male rat offspring exposed to a high protein-to-carbohydrate ratio in the maternal diet were also characterized by elevated BP [55]. In addition, high salt consumption during gestation and lactation has also been associated with hypertension in the offspring in a rat model [40]. However, little is known about the use of large animals to evaluate nutritional programming-induced hypertension.

Worthy of note is that nutritional programming can also be advantageous. Several nutritional interventions have proven to be effective in preventing the development of many adult diseases, including hypertension, with the use of animal models [56]. Since all nutrients during pregnancy play a crucial role in fetal growth and development, studies utilizing animal models of nutritional programming will lead to a better understanding of the timing, optimal dose, and intake duration of nutritional interventions for clinical practice.

### 3.2. Maternal Illnesses and Conditions

Maternal illnesses and complications during pregnancy can cause fetal programming and increase the risk of developing hypertension in offspring. Thus, animal models that mimic chronic illnesses and pregnancy complications have been established to study hypertension of developmental origins. Table 1 shows that rats are the most commonly used animal species. Diverse animal models resembling human illnesses and pregnancy complications have been evaluated, such as hypertensive disorders of pregnancy [57,58], preeclampsia [59,60,61], chronic kidney disease [62], diabetes [63,64], polycystic ovary syndrome [65], maternal inflammation [66,67], maternal hypoxia [68,69], and sleep disorder [70,71].

Hypertensive disorders affect around 10% of pregnancies, which includes the 3–5% of all pregnancies complicated by preeclampsia [72]. A previous cohort study showed that there is an association between maternal hypertension and adverse cardiometabolic outcomes in offspring at 40 years of age, including a 67% increased risk of hypertension [73]. Studies in two animal models—spontaneously hypertensive rat (SHR) and renovascular hypertensive rat—support an association between maternal hypertension and rising BP in the offspring during young adulthood [57,58]. Several animal models have been established that mimic changes in maternal preeclampsia. For example, pregnant rats were administered suramin [59] or N^G^-nitro-L-arginine-methyl ester (L-NAME, an inhibitor of nitric oxide synthase) [60], or underwent a reduced uterine perfusion procedure [61], resulting in elevated BP in their adult offspring. Pregnant women with chronic kidney disease (CKD) are at risk of adverse outcomes for themselves and their offspring [74]. An adenine-induced maternal CKD model was used to study uremia-related adverse outcomes in pregnancy and offspring, including hypertension of developmental origins [59].

Epidemiological observations have established that exposure to gestational diabetes mellitus in utero leads to a high risk of high BP in childhood [75,76]. Hypertension in offspring induced by maternal diabetes is also demonstrable in animal models [63,64]. Although many models have been used for diabetes research [77], only streptozotocin (STZ)-induced diabetes has been modelled for hypertension of developmental origins [63,64]. Both type 1 and type 2 diabetes can be induced by STZ when given to adult [63,64] or neonate rats [63]. Another common pregnancy complication is iron-deficiency anemia. A previous report demonstrated that adult offspring of both sexes in a rat model of maternal iron deficiency had hypertension at 16 weeks of age [36].

Additionally, polycystic ovary syndrome (PCOS), inflammatory disorders, and hypoxia are associated with an increased risk of maternal pregnancy complications [78,79]. In the case of PCOS, the fetus is exposed to high levels of testosterone from the maternal circulation [80]. Thus, a model of maternal hyperandrogenemia by testosterone cypionate administration in pregnant rats in late gestation was developed to study BP in adult offspring [65]. As a result, female offspring exposed to prenatal androgen developed hypertension at 120 days of age [65]. Prenatal exposure to two pyrogens, LPS and zymosan, has been used to mimic maternal inflammation, and both models showed elevated BP in adult offspring [66,67]. Likewise, hypertension can be programmed by prenatal hypoxia in rats [68] or sheep [69]. Moreover, sleep disorders or chronodisruption in pregnant women could have harmful consequences for their offspring, as we reviewed elsewhere [81]. Table 1 shows that adult rat offspring exposed to maternal sleep restriction or constant light prenatally were found to develop hypertension [70,71]. Based on evidence gathered from the above-mentioned studies, various maternal illnesses and conditions indeed impact the offspring’s BP and validate the epidemiological observations. However, whether other maternal conditions such as depression are relevant to the developmental programming of hypertension has not yet been adequately addressed.

It is noteworthy that most animal models employ rats and may evaluate short-term but not long-term outcomes in offspring. Research on DOHaD should now be intensified to validate the observed effects, with long-term follow-up studies using different species to identify the underlying common mechanisms.

### 3.3. Chemical and Medication Exposure

In addition to maternal conditions, early-life chemical and medication exposure has been associated with the developmental programming of hypertension. Table 2 illustrates that prenatal exposure to 2,3,7,8-tetrachlorodibenzo-p-dioxin (TCDD) or bisphenol A leads to increased BP in adult rat offspring [82,83,84]. These findings support the epidemiological data indicating that exposure to environmental chemicals such as endocrine-disrupting chemicals (EDCs) during critical developmental stages can increase the risk of cardiovascular disease later in life [85].

Substance abuse is also a major maternal insult; about 6–16% of pregnant women in the United States are alcohol users, cigarette smokers, or illicit drug users [86]. Previous reports on animal models demonstrated that maternal nicotine, alcohol, or caffeine exposure caused elevated BP in rat offspring [87,88,89]. However, similar models using large animals are not applied at the present time.

Additionally, medication use during pregnancy is also involved in the pathogenesis of programmed hypertension. As shown in Table 2, cyclosporine [90], gentamicin [91], minocycline [92], tenofovir [93], or glucocorticoid [94,95,96,97,98] administration in critical periods of development has been reported to induce hypertension of developmental origins in offspring. Unlike in humans, renal development in rodents continues up to postnatal week 1–2. Thus, adverse events during gestation and the early lactation period can impair nephrogenesis and reduce nephron numbers, resulting in hypertension in later life [99]. Cyclosporine, gentamicin, and glucocorticoid have been related to renal programming and reduced nephron numbers in various animal models [99]. Particularly noteworthy is glucocorticoid, the most extensively studied medication in animal models of programmed hypertension. A developing fetus is prone to being exposed to excessive glucocorticoids through excess maternal corticosteroid use (e.g., due to a stressed pregnancy) or through exogenous administration (e.g., during preterm birth). In rats, both maternal and neonatal administration of dexamethasone induced hypertension in adult offspring [94,95,96]. Likewise, prenatal glucocorticoid administration in a sheep model caused increased BP in the offspring [97,98]. Moreover, the use of minocycline, a tetracycline antibiotic, during pregnancy and lactation was shown to induce programmed hypertension in rat offspring, coinciding with alterations of the gut microbiota and its derived metabolites [92]. Tenofovir, an antiviral drug, can also program hypertension in a rat model [93]. To sum up, different classes of medications contribute to developmental programming of hypertension. It is possible that various insults can cause similar adult phenotypes that converge on common mechanisms, culminating in the development of hypertension.

**Table 2:** Summary of animal models of the developmental programming of hypertension, categorized according to chemical and medication exposure.

Chemical or Medication	Animal Models	Species/Gender	Age at Hypertension Development	Ref.
TCDD	Oral administration of 200 ng/kg TCDD on gestational days 14 and 21 and postnatal days 7 and 14	SD rat/M	12 weeks	[82]
	Oral administration of 200 ng/kg TCDD on gestational days 14 and 21 and postnatal days 7 and 14	SD rat/M	16 weeks	[83]
Bisphenol A	Oral administration of 50 μg/kg/day bisphenol A during pregnancy and lactation	SD rat/M	16 weeks	[84]
Nicotine	Nicotine administration via osmotic mini-pump at 4 μg/kg/min from gestational day 4 to postnatal day 10	SD rat/M	8 months	[87]
Alcohol	Ethanol 1 g/kg by oral gavage on gestational days 13.5 and 14.5	SD rat/M,F	6 months	[88]
Caffeine	Subcutaneous injection of 20 mg/kg caffeine daily during pregnancy	C57BL/6 mouse/M	3 months	[89]
Cyclosporine	Cyclosporine 3.3 mg/kg from gestational day 10 to postnatal day 7	SD rat/M	11 weeks	[90]
Gentamicin	Subcutaneous injection of 110 mg/kg gentamicin from gestational day 10 to 15 or 15 to 20	SD rat/F	1 year	[91]
Minocycline	Minocycline 50 mg/kg via oral gavage during pregnancy and lactation	SD rat/M	12 weeks	[92]
Tenofovir	Tenofovir 100 mg/kg diet from 1 week before mating and during pregnancy	Wistar rat/M	6 months	[93]
Glucocorticoid	Intraperitoneal injection of 0.2 mg/kg dexamethasone on gestational days 15 and 16	SD rat/M	12 weeks	[94]
	Intraperitoneal injection of 0.1 mg/kg dexamethasone from gestational day 16 to 22	SD rat/M	12 weeks	[95]
	Intraperitoneal injection of 0.5 mg/kg dexamethasone on postnatal day 1, 0.3 mg/kg on day 2, and 0.1 mg/kg on day 3.	SD rat/M	12 weeks	[96]
	Intramuscular injection of 0.17 mg/kg betamethasone on gestational days 80 and 81	Sheep/M,F	18 months	[97]
	Intravenous treatment with 0.48 mg/h dexamethasone for 48 h on gestational day 27	Sheep/M,F	16 months	[98]

Studies tabulated according to type of chemical or medication, animal model, and age at evaluation. TCDD = 2,3,7,8-tetrachlorodibenzo-p-dioxin; SD = Sprague-Dawley.

Emerging evidence supports a “two-hit” hypothesis that explains the developmental programming of adult diseases [8]. Hypertension can develop with two sequential hits, the first hit being the response to a prenatal insult, followed by the second hit in response to ongoing programming induced by the first hit. During fetal development, the first hit can lead to morphological changes and functional adaption of vital organ systems, which alone is not sufficient to alter the adult phenotype. Another type of insult may act as a second hit, during which the same mechanism is targeted and could unmask or amplify the underlying defects culminating in a disease state. Accordingly, a number of two-hit models have been used to evaluate whether two distinct hits affect offspring outcomes synergistically or differently when combined as compared to either hit alone. For example, models of a high-fructose diet and TCDD exposure [82], TCDD plus dexamethasone exposure [83], combined bisphenol A and a high-fat diet [84], and a high-fructose diet plus a post-weaning high-fat diet [100] have been established to study hypertension of developmental origins. Together, these animal models have provided evidence of a number of common mechanisms behind hypertension of developmental origins, which will be discussed in turn.

## 4. Common Mechanisms Underlying Hypertension of Developmental Origins

In view of the fact that diverse early-life insults create very similar outcomes in adult offspring, there might be some common mechanistic pathways contributing to the pathogenesis of hypertension of developmental origins. So far, the proposed mechanisms include oxidative stress, aberrant RAS, reduced nephron numbers, gut microbiota dysbiosis, and sex differences [10,18,19,20,21].

### 4.1. Oxidative Stress

During fetal development, overproduction of reactive oxygen species (ROS) under adverse conditions in utero prevails over the defensive antioxidant system, resulting in oxidative stress damage [101]. There are several types of early-life insults linked to oxidative stress in mediating hypertension of developmental origins, including maternal caloric restriction [28,29], a zinc-deficient diet [37], a methyl-donor diet [39], high fat intake [50], high-fructose consumption [51], preeclampsia [60,61], maternal CKD [62], gestational diabetes [63], maternal hypoxia [68,69], TCDD exposure [83], bisphenol A exposure [84], nicotine exposure [87], and glucocorticoid use [94].

Reported mechanisms behind oxidative stress-induced hypertension of developmental origins consist of increased ROS generation [61], decreased antioxidant capacity [35], impaired nitric oxide (NO) signaling pathway [33,59,62,94], and increased oxidative damage [29,82,84,94]. Markers of lipid peroxidation such as malondialdehyde (MDA) and F2-isoprostanes were proven to be elevated in animal models of programmed hypertension induced by a maternal low-protein diet [35], maternal L-NAME administration [60], and reduced uterine perfusion [61]. Additionally, the expression of 8-hydroxydeoxyguanosine (8-OHdG), an oxidative DNA damage marker, was increased in animal models of hypertension programmed by a maternal methyl-donor diet [39], prenatal dexamethasone plus TCDD exposure [82], combined high-fat diet and bisphenol A exposure [84], prenatal dexamethasone exposure [95], and a maternal high-fructose diet [102].

Conversely, many natural and synthetic antioxidants have been used as a reprogramming strategy to prevent hypertension of developmental origins in diverse of animal models [20,103]. These observations suggest the notion that the developmental programming of hypertension might be driven by oxidative stress.

### 4.2. Aberrant Renin-Angiotensin System

Blood pressure is tightly controlled by the renin-angiotensin system (RAS) [104]. The blockade of the RAS provides the rationale for current antihypertensive therapies. The kidney is a major target for all components of the RAS. During kidney development, constituents of the RAS are highly expressed and play key roles in mediating proper renal morphology and physiological function [105]. In humans, RAS blockers have been avoided for pregnant women due to fetopathy and renal maldevelopment [106]. The adult progeny of animals that are transgenic for RAS genes or received angiotensin receptor blocker (ARB) during the nephrogenesis stage to block the RAS have a concurrent reduction in nephron numbers and hypertension [107,108].

An increasing number of animal models related to aberrant RAS are now being developed to evaluate hypertension of developmental programming [109]. Various nutritional insults can program the kidney and RAS concurrently—protein restriction [34], calorie restriction [30], a high-fructose diet [51], and a high-fat diet [110]—resulting in hypertension in adult offspring.

Adult rat offspring of diabetic mothers developed hypertension coinciding with increased angiotensin-converting enzyme (ACE) activity [111]. Other maternal illnesses and conditions such as hypertension [58], CKD [62], chronodisruption [78], and preeclampsia [60] also interfere with aberrant RAS and programmed hypertension. Moreover, programmed hypertension coinciding with dysregulated RAS can be triggered by maternal exposure to TCDD [83], caffeine [89], minocycline [92], or glucocorticoid [94,98].

On the other hand, reprogramming strategies targeting the RAS to prevent hypertension of developmental origins have been employed in various animal models [109]. So far, several early-life interventions have been demonstrated, including renin inhibitor [112], ACE inhibitor [113], ARB [114], and ACE2 activator [115]. Overall, the findings suggest that the interplay between the RAS and other mechanisms in early life is implicated in renal programming and consequently, hypertension in adulthood.

### 4.3. Reduced Nephron Numbers

A nephron is the basic unit of the kidney; however, there are large individual differences in the number of nephrons, ranging from 0.25 to 1.1 million per human kidney [116]. Epidemiologic studies have associated low birth weight and prematurity with low nephron numbers as risk factors for hypertension in later life [117]. Reduced nephron numbers can cause compensatory glomerular hyperfiltration and glomerular hypertension, consequently leading to further nephron loss later in life. Therefore, reduced nephron number has been considered as a key mechanism behind renal programming [118]. Likewise, animal studies have indicated that there are vulnerable periods during kidney development that could lead to a reduced nephron endowment.

In rats, adult offspring develop hypertension coinciding with reduced nephron numbers in response to diverse environmental insults during kidney development. These animal models of renal programming involved maternal exposure to cyclosporine [90], gentamicin [91], or glucocorticoid [94], or maternal diabetes [63], a low-protein diet [119], inflammation [120], or hypoxia [121]. However, reduced nephron numbers per se would not be essential for hypertension of developmental origins and renal programming [118]. The role of altering the nephron number in hypertension of developmental origins is still awaiting discovery but is certainly a subject of great interest.

### 4.4. Gut Microbiota Dysbiosis

Recent evidence suggests that early development of the gut microbiota may impact the programming of adult diseases, including hypertension [122,123]. During gestation, diet-gut microbiota interactions can alter global histone acetylation and methylation, not only in the mother but also in the fetus via contact with her metabolites [124]. Several mechanisms that link gut microbiota dysbiosis to hypertension have been proposed, including increased sympathetic activity, NO inhibition, aberrant RAS, and altered microbial metabolites, such as short-chain fatty acids (SCFAs) [125].

Data from many animal models indicate that gut microbiota dysbiosis may be involved in the developmental programming of hypertension. Various rat models of maternal insults such as hypertension [57], CKD [62], PCOS [65], TCDD exposure [82], minocycline use [92], a high-fructose diet [102], and a high-fat diet [126] have been examined with regard to the impact of gut microbiota dysbiosis on hypertension of developmental origins.

Worth noting is the consumption of probiotics or prebiotics, which has become one dietary strategy for modulating the gut microbiota. Our prior studies reported that maternal consumption of a high-fructose or high-fat diet induced hypertension in adult offspring, which can be prevented by modulating the gut microbiota through the intake of prebiotic inulin or probiotic *Lactobacillus casei* [127,128]. Despite recent studies showing that microbiota-targeted therapies can be applied to various diseases [129], their role in hypertension of developmental origins, especially their use in gestation, awaits further exploration.

### 4.5. Sex Differences

There is a considerable body of literature indicating that sex-dependent differences exist in hypertension of developmental origins [130,131]. It has long been observed that male offspring are more prone to hypertension than female offspring [25,130,131]. Additionally, several mechanisms mentioned above, such as oxidative stress [132], the RAS [133], and gut microbiota [134], are known to respond to environmental stimuli in a sex-specific manner.

Some early-life insults, such as maternal caloric restriction [27], low-protein diet [55], high-fat diet [110], or high-fructose diet [112], or prenatal dexamethasone exposure [135], have been reported to induce hypertension in male but not female offspring. This difference has led many researchers to investigate only males instead of both sexes, as listed in Table 2.

In a prenatal dexamethasone exposure model [135], we found that glucocorticoid-programmed hypertension developed in male but not in female adult offspring. We also observed the absence of hypertension in female offspring coinciding with lower *Agt* mRNA expression, suggesting that sex-dependent renal programming within the RAS may underlie the pathogenesis of programmed hypertension. Additionally, we found that the renal transcriptome is sex-specific in hypertension in offspring programmed by a maternal high-fructose diet [112]. One possible protective mechanism of females being refractory to high-fructose-diet-induced programmed hypertension is related to sex differences in the renal transcriptome. However, whether the increased female sensitivity to insult is beneficial or harmful to the developmental programming of various organs in female fetuses remains unclear. Thus, a better understanding of the sex-dependent mechanisms that underlie hypertension of developmental origins will aid in developing a novel sex-specific strategy to prevent programmed hypertension across genders.

### 4.6. Others

Other molecular mechanisms relevant to the developmental programming of hypertension are evaluated in different animal models, such as impaired sodium transport [10], dysregulated nutrient-sensing signaling [136], increased sympathetic nerve activity [137], and epigenetic regulation [138].

These observations suggest that there might be considerable interplay among the common mechanisms behind the pathogenesis of hypertension of developmental origins, even though this remains speculative. Although numerous mechanisms are outlined above, attention will need to be focused on exploring other potential mechanisms and validating them in different types of animal models. A better understanding of the mechanisms behind hypertension of developmental origins is the key to developing novel reprogramming interventions for further clinical translation.

## 5. Moving Forward: Promising Prospects of Early-Life Interventions

Given the advances in our understanding of the DOHaD research field, it has become apparent that early-life interventions can reprogram molecular mechanisms behind hypertension of developmental origins to prevent the development of hypertension in adulthood. Animal models have been essential in providing ideal reprogramming strategies. As described earlier, many antioxidants have been used as reprogramming strategies to prevent hypertension in offspring in a number of animal models [20,103]: L-arginine [139], L-taurine [140], L-citrulline [60], vitamin C [69], vitamin E [28], folic acid [141], selenium [28], melatonin [39,60,71], resveratrol [83,84,102], and N-acetylcysteine [57,59,60,95]. 

Additionally, several lines of evidence support the idea that early-life interventions targeting specific signaling pathways are of benefit in the prevention of developmental hypertension. First, reprogramming strategies targeting the NO pathway in early life have been employed in various animal models to prevent the development of hypertension in adult progeny. These interventions include supplementation of NO substrate [142], agents that lower asymmetric dimethylarginine (ADMA, an inhibitor of NOS) [95], NO donors [143], and enhancement of NOS expression [144], as reviewed elsewhere [142]. Second, several RAS-based interventions have also shown benefits in protecting against programmed hypertension, such as renin inhibitor, ACEI, ARB, and ACE2 activator [109]. Third, the reprogramming effects of hydrogen sulfide (H_2_S)-based interventions have been shown in diverse animal models [145]. Currently available reprogramming interventions targeting the H_2_S pathway are L-cysteine [146], D-cysteine [146], NAC [147], sodium hydrosulfide [148], and garlic [126]. Finally, the targeting of nutrient-sensing signals such as cyclic adenosine monophosphate-activated protein kinase (AMPK) or peroxisome proliferator-activated receptor (PPAR) has been noted to regulate downstream target genes, thereby reprogramming hypertension induced by various maternal insults [149,150,151,152,153,154]. This review provides a general overview of the various early-life interventions that show benefits with regard to hypertension of developmental origins. Despite the tremendous advances made from animal research, their clinical translation is still a long way off.

## 6. Selection of Appropriate Animal Models to Study Hypertension of Developmental Origins

### 6.1. Important Issues for Consideration

Even though significant advances have been made in developing diverse animal models to study hypertension of developmental origins, the need for meaningful clinical translation remains a research priority. The following conditions should be taken into consideration when we select animal models. First, the timing of the animal’s organogenesis is similar to that of humans. Second, the gestation period and litter size should be comparable to those of humans. Third, it is crucial that animal models share similar features of adverse outcomes to those seen in human studies, which can be measured. Finally, any effective therapeutic intervention must be evaluated and validated.

### 6.2. Timing of Organogenesis

Across different species, critical development periods for major organ systems are not uniform. Blood pressure is tightly controlled by coordination among the kidney, heart, brain, and other organ systems. As such, the translatability of studies performed in animals should be approached with caution, as many key stages of BP-controlled organ development that occur before birth in humans occur after birth in some species [103,136,137,155].

Many animal studies on hypertension of developmental origins focus on renal programming [118,137]. Kidney development starts at week 3 and ceases at approximately 36 weeks of gestation in humans [156]. Unlike in humans, rat kidneys continue to develop after birth and complete at 1 to 2 weeks postnatally [157]. Accordingly, adverse environmental conditions during pregnancy as well as lactation can impair kidney development, consequently resulting in hypertension in rodents [118]. For example, repeated dexamethasone administration on embryonic days 15 and 16 [94], from gestational days 16 to 22 [95], or from postnatal days 1 to 3 [96] was associated with developmental programming of hypertension in adult rat offspring.

Another unsolved problem is that almost no studies have taken a comprehensive approach to simultaneously evaluating every BP-controlled organ system in response to in utero exposure at specific developmental stages to assess their relative vulnerability in an experiment. Due to the complex nature of the interplay between organogenesis and environmental insults, the programming effect on various organs might be dissimilar in different animal species. Hence, it is apparent that the selected animal paradigm should mirror the timing of human organ development as closely as possible so that the effects of early-life insults can be fully assessed.

### 6.3. Gestation Period and Litter Size

The advantages of a shorter gestation period and higher offspring yield compared to large animal models make rodent models the most commonly used in DOHaD research. There is a large set of studies on hypertension of developmental origins that were carried out in rats (Table 1 and Table 2). The average gestation period for rats is within 23 days, compared to 280 days for humans [158]. If an early-life insult is induced by surgical manipulation or if delivery requires repeated procedures, short gestation in rodents could become disadvantageous. In addition, the short gestation time may not allow for the permanent resolution of developmental plasticity and the identification of critical time periods that are vulnerable to insults.

Unlike humans, rodents generally have more than one offspring, and litter sizes of 8–12 pups are usually seen. Such a large litter size is also a disadvantage when compared to singleton births common in humans and large animal models. Accordingly, normalizing the size of each litter after birth should be considered to control for differences in offspring food intake, maternal care, and pup growth [159]. Since these limitations exist, the complete translation of findings in rodents to human medicine is seriously compromised.

On the other hand, gestational length in sheep is around 150 days, during which the fetal size and development rate are similar to those of humans [160]. With the use of ewe models, maternal caloric restriction [30], maternal hypoxia [69], and prenatal glucocorticoid exposure [97,98] have been shown to cause hypertension in adult progeny. Although these early insults have shown the same adverse effects on offspring BP in sheep and rats, whether different gestation periods and litter sizes differentially impact hypertension of developmental origins in rats and large animals awaits further evaluation.

### 6.4. Outcome Measurements

As we mentioned earlier, rats are the most commonly used species for the developmental programming of hypertension. However, a critical assessment of the data show that this phenomenon is mostly observed when BP is typically measured by the tail cuff method; in contrast, hypertension is not detected in telemetrically instrumented animals [161]. Although BP data obtained from the tail cuff method are reported to correlate well with findings of direct arterial catheter and telemetry methods [162], part of the increased BP in offspring found after early-life insults may be due to an increased stress response related to sympathetic nerve activity.

In adulthood, one rat month is roughly equivalent to three human years [158]. Accordingly, Table 1 lists the timing of hypertension development measured in rats from 12 weeks to 8 months of age, which is equivalent to humans of a specific age group ranging from childhood to early adulthood. Thus, there remain gaps in our knowledge regarding the long-term adverse effects of maternal insults on BP in older adult offspring.

Several species have been studied for cardiovascular outcomes programmed by maternal adverse exposure, including guinea pigs [163], swine [164], and non-human primates [165]. However, none of them have been used to study hypertension of developmental origins. It is important to remember that large animals should not be neglected, as they are generally more physiologically suitable models with regard to human conditions.

In the current review, the wide range of early-life insults certainly influenced the outcomes, resulting in the reported heterogeneity. The results depended strongly on the applied measurement technique and animal model. Methodological heterogeneity is another reason for the observed heterogeneity. A huge percentage of studies employed male-only small animal models with small sample sizes. Future animal studies should improve the methodological quality by applying randomization, blinding, and sample size calculation techniques in order to avoid bias and collect data of better quality.

### 6.5. Effective Interventions

Currently, reprogramming strategies could be categorized as nutritional intervention, lifestyle modification, or pharmacological therapy. It stands to reason that avoiding in utero exposure to adverse conditions is the most effective strategy for preventing hypertension of developmental origins. Another approach is the use of nutritional intervention during pregnancy and lactation [56]. Although the targeting of specific nutrients as a reprogramming strategy opens a new avenue for prevention [25], there remains a lack of accurate dietary recommendations for specific nutritional requirements for pregnant women in case of deficiencies [166,167].

Research on short-lived rodent models has provided significant results, revealing potential pharmacological therapies for preventing hypertension of developmental origins. However, disparities in the therapeutic doses, timing and duration, and animal models used are among the major concerns. The standardization of animal experiments will improve the comparability of such studies. During the preparation of the current review, we found that almost no studies tested different doses or the use of different species. Additionally, the follow-up period after the cessation of interventions in most cited studies was rather short.

The efficacy of the intervention can be influenced by its duration with respect to organ development in a dose- and species-specific manner. Thus, further translational research into the pharmacokinetics and metabolism of pharmacological intervention is required to validate and compare its safety and therapeutic potential between humans and other species.

## 7. Conclusions and Future Perspectives

Various small (e.g., rat and mouse) and large (e.g., cow and sheep) animal models have made important contributions to the DOHaD research field, giving rise to convincing evidence of a causal relationship between various early-life insults and the risk of developing hypertension in later life. Our review highlights that animal models are not only used to investigate the mechanisms behind hypertension of developmental origins, but also have an impact on the development of early-life interventions as a reprogramming strategy to prevent the development of hypertension in adulthood.

There are still several questions that need to be answered. In the last decades, many insults have been identified by epidemiological and animal studies. Nevertheless, there is a growing need to identify all factors that can adversely impact the BP of offspring. Additionally, this review did not consider the potential for the programming of hypertension by paternal factors that clearly exist in the DOHaD field [168]. Moreover, little reliable information currently exists with regard to optimal doses and durations of pharmacological interventions for pregnant women and the long-term effects on their offspring. Currently, preventive strategies should focus on avoiding exposure to theoretically harmful agents perinatally and promoting a healthy lifestyle.

Each of the abovementioned animal models was used to study a specific hypothesis and neither can be considered superior with regard to all aspects of research on hypertension of developmental origins. Therefore, further research is needed to gain a better understanding of the types of early-life insults, other mechanisms behind hypertension of developmental origins, the ideal therapeutic dose and duration of early intervention, and the appropriate animal species. It is proposed that taking a DOHaD approach with maximum use of the animal evidence should be of benefit in reducing the global burden of hypertension.

## Figures and Tables

**Table 1:** Summary of animal models of the developmental programming of hypertension, categorized according to maternal illness and pregnancy complications.

Maternal Illnesses and Conditions	Animal Models	Species/Gender	Age at Hypertension Development	Ref.
Hypertensive disorders of pregnancy	Genetic hypertension model	SHR/M	12 weeks	[57]
	2-kidney, 1-clip renovascular hypertension model	SD rat/M,F	16 weeks	[58]
Preeclampsia	Intraperitoneal administration of 60 mg/kg suramin on gestational days 10 and 11	SD rat/M	12 weeks	[59]
	Subcutaneous administration of 60 mg/kg L-NAME during pregnancy	SD rat/M	12 weeks	[60]
	Reduced uterine perfusion	SD rat/M	16 weeks	[61]
Chronic kidney disease	0.5% adenine supplementation from 3 weeks before pregnancy until 3 weeks after delivery	SD rat/M	12 weeks	[62]
Type 1 diabetes	Single intraperitoneal injection of 45 mg/kg STZ on gestational day 0	SD rat/M	12 weeks	[63]
	Single intraperitoneal injection of 35 mg/kg STZ on gestational day 0	SD rat/M	6 months	[64]
Type 2 diabetes	Mother rat received single intraperitoneal injection of 50 mg/kg STZ at newborn stage	SD rat/M	12 weeks	[63]
Anemia	Iron-deficiency diet from 4 weeks before pregnancy until delivery	Rowett hooded Lister rat/M & F	16 weeks	[36]
Polycystic ovary syndrome	Subcutaneous injection of 5 mg/kg testosterone cypionate on gestational day 20	Wistar rat/F	120 days	[65]
Maternal inflammation	Intraperitoneal administration of 0.79 mg/kg LPS on gestational days 8, 10, and 12	SD rat/M & F	12 weeks	[66]
	Intraperitoneal injection of 2.37 mg/kg zymosan on gestation days 8, 10, and 12	SD rat/M	66 weeks	[67]
Maternal hypoxia	Hypoxia maintained at constant inspired fraction of oxygen of 13% from gestational day 6 to 20	Wistar rat/M	4 months	[68]
	Hypoxia maintained at 10% oxygen from gestational day 105 to 145	Sheep/F	9 months	[69]
Sleep disorder	Sleep restriction	Wistar rat/M	3 months	[70]
	24 h constant light exposure during pregnancy	SD rat/M	12 weeks	[71]

Studies tabulated according to types of maternal illnesses and conditions, animal model, and age at evaluation. L−NAME = N^G^-nitro-L-arginine-methyl ester; STZ = streptozotocin; LPS = lipopolysaccharide; SHR = spontaneously hypertensive rat; SD = Sprague-Dawley.

## Data Availability

Data will be available upon request.
